# Metabolomic Analysis of *Aspergillus niger* Isolated From the International Space Station Reveals Enhanced Production Levels of the Antioxidant Pyranonigrin A

**DOI:** 10.3389/fmicb.2020.00931

**Published:** 2020-05-21

**Authors:** Jillian Romsdahl, Adriana Blachowicz, Yi-Ming Chiang, Kasthuri Venkateswaran, Clay C. C. Wang

**Affiliations:** ^1^Department of Pharmacology and Pharmaceutical Sciences, School of Pharmacy, University of Southern California, Los Angeles, CA, United States; ^2^Biotechnology and Planetary Protection Group, Jet Propulsion Laboratory, California Institute of Technology, Pasadena, CA, United States; ^3^Department of Chemistry, Dornsife College of Letters, Arts, and Sciences, University of Southern California, Los Angeles, CA, United States

**Keywords:** secondary metabolites, *Aspergillus niger*, International Space Station, pyranonigrin A, antioxidant

## Abstract

Secondary metabolite (SM) production in *Aspergillus niger* JSC-093350089, isolated from the International Space Station (ISS), is reported, along with a comparison to the experimentally established strain ATCC 1015. The analysis revealed enhanced production levels of naphtho-γ-pyrones and therapeutically relevant SMs, including bicoumanigrin A, aurasperones A and B, and the antioxidant pyranonigrin A. Genetic variants that may be responsible for increased SM production levels in JSC-093350089 were identified. These findings include INDELs within the predicted promoter region of *flbA*, which encodes a developmental regulator that modulates pyranonigrin A production via regulation of Fum21. The pyranonigrin A biosynthetic gene cluster was confirmed in *A. niger*, which revealed the involvement of a previously undescribed gene, *pyrE*, in its biosynthesis. UVC sensitivity assays enabled characterization of pyranonigrin A as a UV resistance agent in the ISS isolate.

## Introduction

Filamentous fungi are ubiquitous in spacecraft environments due to anthropogenic contamination and an inability to completely sterilize the craft and cargo (Pierson, 2001; Novikova et al., 2006; Van Houdt et al., 2012). Microbial infections are a major health risk for astronauts, and are exacerbated by the combined stresses of microgravity, sleep disruption, alterations in food intake, confined living space, and high levels of radiation that can compromise the immune system (Pierson, 2001; Sonnenfeld et al., 2003). Additionally, several studies have indicated that spacecraft environments increase microbial virulence and antimicrobial resistance (Tixador et al., 1985; Wilson et al., 2007, 2008). As we make strides toward human interplanetary exploration, investigations into the characteristics of filamentous fungi that reside in spacecraft environments are critical for crew health. Additionally, such studies present diverse industrial and therapeutic opportunities, as fungi produce a plethora of bioactive secondary metabolites (SMs) in response to external stressors. These small molecules often kill or inhibit growth of other organisms, enabling fungi to successfully compete within the complex ecosystem they reside in. While some SMs are toxic to humans, others have diverse industrial and therapeutic applications, including antibiotic, anticancer, antioxidant, immunosuppressant, and cholesterol-lowering activities (Newman and Cragg, 2012). The remarkable structural and functional diversity of fungal SMs arise from the combinatorial and modular nature of their biosynthesis, in which the SM core backbone is biosynthesized by a core synthase enzyme, such as a polyketide synthase (PKS), a nonribosomal peptide synthetase (NRPS), or a hybrid PKS-NRPS, and further diversified by a number of tailoring enzymes that are clustered together within the genome (Fischbach and Walsh, 2006).

In order to understand the characteristics of microbes residing in the International Space Station (ISS), National Aeronautics and Space Administration (NASA) has implemented a robust microbial monitoring system (Pierson et al., 2013). The filamentous fungus *Aspergillus niger* has been reported to be a predominant ISS isolate (Checinska et al., 2015), and is frequently detected in other built environments (Haleem Khan and Mohan Karuppayil, 2012; Varga et al., 2014). *A. niger* is a melanized fungal species commonly used in the biotech industry as a production host for citric acid and enzymes (Schuster et al., 2002). Melanized fungi are commonly isolated from highly irradiated environments (Singaravelan et al., 2008; Selbmann et al., 2015), and it has been reported that the electronic properties of melanin change following exposure to ionizing radiation (Dadachova et al., 2007). Additionally, several studies have reported the association of melanin production with fungal virulence (Kwon-Chung et al., 1982; Wang et al., 1995; Nosanchuk et al., 1998). Together, these findings demonstrate the need for studies that assess the characteristics of melanized fungi inhabiting spacecraft environments.

Naphtho-γ-pyrones are the predominant class of SMs produced by *A. niger*, and they possess a diverse array of reported biological properties, including anti-HIV, anti-hyperuricemia, anti-tubercular, antimicrobial, antitumor, and antioxidant activities (Choque et al., 2015). Advances in genome sequencing of *A. niger* have revealed its capacity to produce many other SMs in addition to the naphtho-γ-pyrones, as its genome harbors 46 PKSs, 35 NRPSs, and 9 NRPS-PKS hybrid genes (Romsdahl and Wang, 2019). Many of these SM biosynthesis genes are silent or expressed at very low levels in standard laboratory conditions, which has resulted in a lack of studies on SMs that may have useful industrial and therapeutic properties. Given that fungi produce SMs in response to environmental stress, investigations into SM production of *A. niger* strains isolated from space environments may reveal enhanced production levels of bioactive molecules and provide insight into the mechanisms underlying such adaptations.

Here, we report the SM production of JSC-093350089, a previously described strain of *A. niger* isolated from surfaces of the International Space Station (ISS) (Romsdahl et al., 2018). Our analysis revealed that the ISS isolate produces high levels of the antioxidant pyranonigrin A (Miyake et al., 2007), along with other bioactive SMs, such as kotanin, pestalamide B, and naphtho-γ-pyrones, when compared to the experimentally established strain ATCC 1015. To investigate differences between the strains that may explain the increased production of some SMs in JSC-093350089, we conducted a comparative genetic analysis of biosynthesis clusters and regulators of differentially produced SMs. Finally, the UV resistance potential of pyranonigrin A was assessed.

## Materials and Methods

### Secondary Metabolite Extraction and Analysis

JSC-093350089 and ATCC 1015 were cultivated at 28°C on GMM agar plates, starting with 1 × 10^7^ spores per Petri dish (*D* = 10 cm). After 5 days, agar was chopped into small pieces and extracted with 25 ml methanol (MeOH), followed by 25 ml 1:1 MeOH-dichloromethane, each with 1 h of sonication and filtration. The extract was evaporated *in vacuo* and re-dissolved in 2 ml of 20% dimethyl sulfoxide in MeOH and a portion (10 μl) was examined by high performance liquid chromatography-photodiode array detection-mass spectroscopy (HPLC-DAD-MS) analysis. HPLC-MS was carried out using a ThermoFinnigan LCQ Advantage ion trap mass spectrometer with a reverse-phase C18 column (3 μm; 2.1 by 100 mm; Alltech Prevail) at a flow rate of 125 μl/min. The solvent gradient for HPLC-DAD-MS was 95% MeCN/H_2_O (solvent B) in 5% MeCN/H_2_O (solvent A) both containing 0.05% formic acid, as follows: 0% solvent B from 0 to 5 min, 0 to 100% solvent B from 5 min to 35 min, 100 to 0% solvent B from 40 to 45 min, and re-equilibration with solvent B from 45 to 50 min.

### Strains and Molecular Manipulations

The *A. niger* wild-type and mutant strains used in this study are listed in Supplementary Table S1, primers used in this study are listed in Supplementary Table S2, and diagnostic PCR results are shown in Supplementary Figure S1. Deletion cassettes were generated using the double joint PCR technique (Supplementary Figure S2; Yu et al., 2004). DNA insertions into the *A. niger* genome were performed using protoplasts and standard PEG transformation. To develop an efficient gene targeting system in JSC-093350089, the *kusA* gene was first deleted by replacing it with the hygromycin resistance marker (*hph*). The two amplified flanking sequences and the hygromycin phosphortransferase gene (*hph)* marker cassette amplified from pCB1003 (Fungal Genetics Stock Center) were fused together into one construct by fusion PCR using nested primers, and the mutation was selected for by growth on media containing 100 μg/ml hygromycin. Diagnostic PCR was performed on the deletant strain using external primers (P1 and P6) from the first round of PCR. The difference in size between the gene replaced by the selection marker and the native gene allowed us to determine whether the transformants carried the correct gene replacement.

Next, an auxotrophic mutant in the *kusA-* background was generated by deleting the *pyrG* gene. Two ∼1500 base pair fragments upstream and downstream of *pyrG* were amplified and fused together (Supplementary Figure S3). The mutation was selected for by growth on media supplemented with 1.5 mg/ml of 5-fluoroorotic acid (5-FOA), as only cells lacking the *pyrG* gene can survive when 5-FOA is present. The correct transformants were identified by performing diagnostic PCR on the deletant strain using external primers (P1 and P6) from the first round of PCR. All other deletant strains were generated by replacing each target gene with the *A. fumigatus pyrG* gene (*AfpyrG*). Double deletion mutants were generated by recycling the *AfpyrG* gene in CW12006. This involved amplifying two ∼1500 base pair fragments upstream and downstream from the *alba*::*AfpyrG* region of JSC-093350089 *albA*Δ genome, which were then fused together. The mutation was selected for by growth on media supplemented with 1.5 mg/ml of 5-FOA. Correct transformants were identified using diagnostic PCR with external and internal primers (P1 and *AfpyrG* rev; *AfpyrG* Fw and P6).

To reintegrate the *kusA* gene into the genome of CW12005, the A*fpyrG* gene used to initially delete *pyrA* was deleted according to the strategy described in Supplementary Figure S3, which generated CW12014. The *kusA* gene was amplified from JSC-093350089 gDNA to include ∼1500 base pair fragment upstream and ∼500 bp fragment downstream from *kusA*. This fragment was then fused to the *AfpyrG* gene and the original 3′ region amplified for initial *kusA* deletion (Supplementary Figure S4). The *kusA* was reintegrated into the genome of CW12014 to generate CW12015. Correct transformants were identified using diagnostic PCR with external primers (P1 and P6).

### Genetic Variant Analysis

Paired-end Illumina sequence reads, obtained from a previous study (Romsdahl et al., 2018), were trimmed using Trimmomatic v 0.36 (Bolger et al., 2014) and quality was checked using FastQC v 0.11.7 (Andrews, 2010). The *A. niger* ATCC 1015 genome and annotation files were downloaded from the FungiDB web portal (Stajich et al., 2012). Trimmed reads were mapped to the ATCC 1015 reference genome using the Burrows-Wheeler Aligner (BWA) software package v 0.7.17 (Li and Durbin, 2009). Mapped read files were further processed using SAMtools v 1.9 (Li et al., 2009) and PCR artifacts were removed using Picard tools MarkDuplicates^1^. Single nucleotide polymorphisms (SNPs) and insertions/deletions (INDELs) were identified using GATK v 3.8.7 (McKenna et al., 2010). Sequence reads containing putative INDELs were realigned using GATK’s IndelRealigner and variants within each sample were called using GATK’s Haplotype Caller. GATK’s VariantFiltration was used to filter the resulting Variant Call Format (VCF) file using stringent cutoffs for quality and coverage {SNPs: QD < 2.0, MQ < 40.0, QUAL < 100, FS > 60.0, MQRankSum < −12.5, SOR > 4.0, ReadPosRankSum < −8.0; INDELs: QD < 2.0, FS > 200.0, MQRankSum < −12.5, SOR > 4, InbreedingCoeff < −0.8, ReadPosRankSum < −20.0}, so that only high-quality variants remained. PromPredict was used to predict if any upstream intergenic variants within 1000 bps of the coding sequence (CDS) occurred within the promoter region (Rangannan and Bansal, 2010; Yella et al., 2018).

### UVC Resistance Analysis

UVC radiation resistance was assessed using JSC-093350089 WT and CW12015. Both strains were cultivated at 28°C on GMM agar plates by seeding 1 × 10^7^ spores per Petri dish (*D* = 10 cm). Spores were collected after 5 days of growth and counted. An equal amount of spores were resuspended in 5 ml of GMM agar and poured onto Petri dishes consisting of 20 ml GMM agar. Mycelia-containing plates were exposed to varying doses of UVC radiation in triplicate using a CL-1000 Ultraviolet Crosslinker (UVP, Inc.).

## Results

### Secondary Metabolite Analysis of JSC-093350089

**FIGURE 1:**
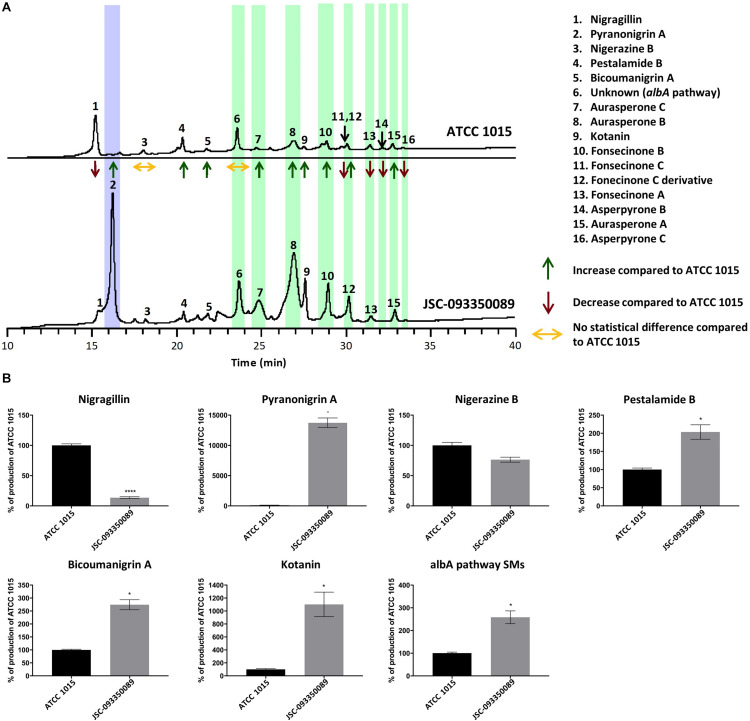
**(A)** Secondary metabolite production in JSC-093350089 relative to ATCC 1015 following growth on GMM for 5 days, as detected by DAD total scan. Each individual metabolite’s production yield is reported as increased, decreased, or no change, compared to that of ATCC 1015. Pyranonigrin A is highlighted in purple and *albA* pathway SMs are highlighted in green. **(B)** Quantification of secondary metabolites showing percent change for each metabolite in JSC-093350089 when compared to ATCC 1015. Significance was determined using Welch’s *t*-test. *P ≤ 0.05; ****P ≤ 0.0001.

SM profiles of JSC-093350089 and ATCC 1015 were examined after growth on GMM agar medium using high-performance liquid chromatography coupled with diode-array detection and electrospray ionization tandem mass spectrometry (HPLC-DAD-MS). SMs were identified based on mass, UV-Vis absorption, and retention time (Supplementary Figure S5), which were in good agreement with literature (Chiang et al., 2011). The identity of pyranonigrin A was further verified by purchasing the pure compound from Enzo Life Sciences (Supplementary Figure S6). The data revealed that each strain produced a distinct SM profile, with the production yield of most SMs significantly altered (Figure 1A). Production yield analysis was carried out for each SM (Figure 1B and Supplementary Figure S7). Compared to ATCC 1015, a significant decrease in the production of nigragillin, an insecticide (Isogai et al., 1975), was observed (*P* = 0.0001). The most significant difference was observed with the antioxidant pyranonigrin A (Miyake et al., 2007), highlighted in purple in Figure 1A, which exhibited a 6000% increase in production in JSC-093350089 (*P* = 0.04), as it was produced at basal levels in ATCC 1015. Nigerazine B displayed no statistical difference in production levels (*P* = 0.06). In the ISS strain, pestalamide B production was approximately 2 times that of ATCC 1015 (*P* = 0.03), and bicoumanigrin, which was reported to have cytotoxic activity against human cancer cell lines (Hiort et al., 2004), exhibited a production yield 2.5 times that of ATCC 1015 (*P* = 0.01). Kotanin production in the ISS strain was approximately 10 times that of ATCC 1015 (*P* = 0.03).

The majority of SMs produced were identified as naphtho-γ-pyrones, including aurasperone A, B, and C, fonsecinone A, B, and C, a fonsecinone C derivative, and asperpyrone B and C. These SMs, highlighted in green in Figure 1A, are biosynthesized by the PKS AlbA (Chiang et al., 2011). The molecular formula of the final SM, labeled as peak 6 in Figure 1A, was predicted using high-resolution mass spectrometry, and a thorough literature search revealed that no known *A. niger* SM matched this formula. This SM was later determined to also be biosynthesized by the *albA* pathway when *A. niger* devoid of AlbA failed to produce the unknown compound. The combined production yields of *albA* pathway SMs were approximately 2.5 times higher in the ISS stain compared to ATCC 1015 (*P* = 0.03).

### Analysis of the Potential Gene Clusters Responsible for Production of Pyranonigrin A *in silico*

To facilitate a more detailed analysis of the underlying genetics that may have led to enhanced production of pyranonigrin A in JSC-093350089, we set out to identify the biosynthetic gene cluster in *A. niger*. First, we searched for the core backbone synthase gene involved in pyranonigrin A biosynthesis. The biosynthetic pathway of pyranonigrin E, a SM very similar to pyranonigrin A, was recently proposed and *pynA* (An11g00250) was identified as the PKS-NRPS hybrid involved in its biosynthesis (Awakawa et al., 2013). We hypothesized that pyranonigrin A is either biosynthesized by the same cluster responsible for pyranonigrin E production, or by a different cluster harboring a PKS-NRPS hybrid gene similar to *pynA*. The genome of ATCC 1015 possesses 8 PKS-NRPS hybrid genes other than *pynA*. BLAST analysis was performed using the Joint Genome Institute (JGI) MycoCosm database (Grigoriev et al., 2014) on the 8 remaining PKS-NRPS hybrids to determine which genes possessed high sequence homology to *pynA*. The results revealed that An18g00520 (*pyrA*) possessed high sequence similarity to *pynA*, with 53.4% sequence identity and 89.8% subject coverage (Supplementary Table S3).

### Development of an Efficient Gene Targeting System in JSC-093350089 and Identification of the PKS-NRPS Hybrid Responsible for Pyranonigrin A Biosynthesis

To determine which of the two putative PKS-NRPS hybrids is involved in the biosynthesis of pyranonigrin A, a genetic system was developed in JSC-093350089. The *kusA* gene was first deleted to decrease the rate of nonhomologous integration of transforming DNA fragments, thereby improving gene targeting efficiency (Meyer et al., 2007). Next, the *pyrG* gene was deleted in the *kusA*- background to generate CW12003, an auxotrophic mutant that requires uracil and uridine supplementation (Arentshorst et al., 2015). CW12003 was then used to generate mutant strains CW12004 and CW12005, which had the *pynA* gene and An18g00520 genes deleted, respectively. JSC-093350089 and the two mutant strains were then cultured on GMM, and SMs were extracted following 5 days of growth and subjected to HPLC-DAD-MS analysis. Observation of SM traces as detected by UV-Vis total scan and mass spectroscopy in positive ion mode [M+H]^+^ *m/z* = 224 revealed pyranonigrin A production in *pynA-* (CW12004) and the complete elimination of pyranonigrin A in An18g00520- (CW12005) (Supplementary Figure S8), indicating that An18g00520 is responsible for the production of pyranonigrin A. This finding was recently confirmed in *Penicillium thymicola* and reported while our work was being completed (Tang et al., 2018).

### Identification of Pyranonigrin A Biosynthesis Gene Cluster

**FIGURE 2:**
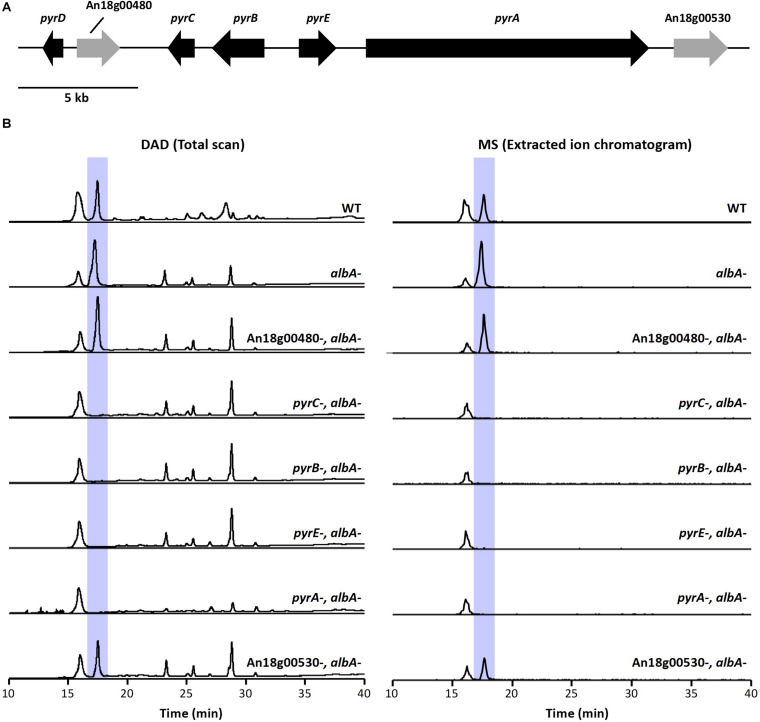
**(A)** Schematic representation of *pyr* cluster. Arrows indicate direction of transcription and the relative size of each open reading frame (ORF). **(B)** DAD total scan and MS extracted ion chromatogram at *m/z* 224 of extracts from the *pyr* mutant library generated in JSC-093350089 with the *albA-* genetic background. Highlighted peaks indicate pyranonigrin A production.

Next, we aimed to identify additional genes involved in pyranonigrin A biosynthesis. To accomplish this, we identified genes surrounding *pyrA* (Figure 2A and Table 1), as the genes involved in fungal SM biosynthesis are usually clustered in the genome (Keller et al., 2005). Interestingly, when we compared the genes surrounding *pyrA* in *A. niger* to their homologs *in P. thymicola* using the JGI MycoCosm database (Grigoriev et al., 2014), we noticed that the distribution of genes surrounding *pyrA* differed between the *P. thymicola* and *A. niger* genomes (Supplementary Figure S9; Tang et al., 2018). For example, *pyrD*, a hydrolase predicted to be involved in pyranonigrin A biosynthesis in *P. thymicola*, is adjacent to *pyrC* in the *P. thymicola* genome and has only two genes separating it from *pyrA*. However, its homolog within the *A. niger* genome, An18g00470, is adjacent to An18g00480 and has four genes separating it from *pyrA*. Similarly, although An18g00510 is adjacent to *pyrA* in the *A. niger* genome, its *P. thymicola* homolog has three genes separating it from *pyrA* in the *P. thymicola* genome, and was predicted to not be involved in pyranonigrin A biosynthesis (Tang et al., 2018). To investigate these observations and identify genes encoding pyranonigrin A biosynthetic tailoring enzymes, we generated a gene deletion library to identify the genes involved in pyranonigrin A biosynthesis.

**TABLE 1:** Putative function of genes within the pyranonigrin A biosynthetic gene cluster and their homologs in *Penicillium thymicola*.

Pyranonigrin A biosynthesis gene	Gene name	*P. thymicola* homolog JGI protein ID	Identity (%)	Putative function
*pyrD*	An18g00470	180933	148/256 (58%)	Hydrolase
	An18g00480	104760	386/554 (70%)	Cyclohexamide resistance protein
*pyrC*	An18g00490	104735	190/269 (71%)	FAD binding monooxygenase
*pyrB*	An18g00500	104701	296/453 (65%)	Cytochrome P450
*pyrE*	An18g00510	168730	326/454 (72%)	FAD binding oxidoreductase
*pyrA*	An18g00520	168734	2371/3881 (61%)	PKS-NRPS hybrid
	An18g00530			Hypothetical protein

*A. niger* produces large quantities of naphtho-γ-pyrone SMs (Chiang et al., 2011), which we suspected may hinder our ability to detect any intermediate compounds in JSC-093350089 tailoring enzyme deletant strains. To circumvent this, we first generated CW12006, a JSC-093350089 mutant strain deficient in AlbA, and therefore also deficient in naphtho-γ-pyrone production. The *AfpyrG* gene was then recycled (Supplementary Figure S3) to enable the generation of additional deletion mutations in CW12007. Next, we used CW12007 to individually delete 5 genes surrounding *pyrA* and generate a pyranonigrin A biosynthetic gene cluster mutant library with *albA-* genetic background. The deletant strains were cultured in pyranonigrin A-producing conditions and their SM production was analyzed using HPLC-DAD-MS (Figure 2B). Deletion strains CW12009, CW12010, and CW12011, which had An18g00490, An18g00500, and An18g00510 deleted, respectively, resulted in the complete elimination of pyranonigrin A, which confirmed their involvement in its biosynthesis. Deletion strains CW12008 and CW12013, which had An18g00480 and An18g00530 deleted, respectively, resulted in unchanged SM profiles, which indicated that these two genes are not involved in pyranonigrin A biosynthesis. These results suggest that the pyranonigrin A biosynthetic gene cluster includes *pyrA*, *pyrB*, *pyrC*, and An18g00510, which we designated as *pyrE* (Figure 2A and Table 1).

These findings are in contrast to the recent study conducted in *P. thymicola*, which did not include *pyrE* in the proposed pyranonigrin A biosynthetic gene cluster (Tang et al., 2018). In this study, heterologous expression of the suspected *pyr* cluster and adjacent genes, which included *pyrE*, led to production of pyranonigrin A. However, *pyrE* (*orf2*) was excluded from the proposed pathway because it was found to not be expressed with the rest of the cluster in pyranonigrin A-producing conditions. Our data indicates that *pyrE* is involved in pyranonigrin A biosynthesis, which as an oxidoreductase may be responsible for the final dehydrogenation or hydroxylation step, which were previously proposed to both be carried out by *pyrB*. This does not exclude the potential involvement of *pyrD* in pyranonigrin A biosynthesis, which was found by Tang et al. (2018) to be co-regulated with *pyrA-pyrC* and was suggested to be involved in tetramic acid formation.

### Genetic Variant Analysis of Clusters Producing SMs That Exhibit Increased Production Levels in JSC-093350089

Having identified the genes responsible for pyranonigrin A production in *A. niger*, we next conducted a genome-level analysis to investigate potential reasons for the enhanced pyranonigrin A production levels in JSC-093350089 relative to ATCC 1015. For this analysis, we also investigated genes involved in the production of kotanin, pestalamide B, and *albA* pathway SMs (aurasperones, fonsecinones, asperpyrones), all of which also featured increased production levels in JSC-093350089 (Figure 1). The biosynthetic gene cluster for kotanin has been identified in *A. niger*, and includes genes encoding non-reducing-PKS (NR-PKS) *ktnS*, O-methytransferase *ktnB*, cytochrome P450 *ktnC*, and flavine-binding monooxygenase *ktnD* (Gil Girol et al., 2012). The biosynthetic gene cluster for pestalamide A was recently elucidated following deletion of the histone acetyltransferase *gcnE*, which led to production of pestalamide A as well as other SMs (Wang et al., 2018). Pestalamide A is a 4*H*-pyran-4-one derivative of pestalamide B, and therefore pestalamide B is likely formed from the same cluster as pestalamide A. This cluster harbors genes encoding NR-PKS *epaA*, ferulate:CoA ligase *epaB*, acyl-CoA transferase *epaC*, oxidoreductase orf1, 3-hydroxybenzoate 4-hydoxylase orf2, and salicylate hydroxylase orf3. Naphtho-γ-pyrones, including the aurasperones, fonsecinones, asperpyrones, are biosynthesized by *albA*, which encodes a PKS that is also crucial for melanin production (Chiang et al., 2011). Tailoring enzymes involved in the biosynthesis of these naphtho-γ-pyrones are not clustered with *albA* and have not been identified.

With this information at hand, we set out to identify SNPs and INDELs occurring in SM cluster regions in JSC-093350089 when compared to ATCC 1015. To accomplish this, JSC-093350089 paired-end reads, obtained from a previous study (Romsdahl et al., 2018), were aligned to the ATCC 1015 reference genome, which resulted in the identification of 5264 SNPs (Supplementary Table S4) and 47446 INDELs (Supplementary Table S5). Variants occurring within the *pyr*, *ktn*, and *epa* clusters and near the *albA* gene are displayed in Table 2. Within the *pyr* cluster, no variants were observed within the coding sequence (CDS) or in the upstream intergenic promoter region. One INDEL was identified downstream of the *pyrA* gene. Two INDEL variations were also observed in *pyrC* introns, and the region downstream of the *pyrC* gene was highly variable compared to ATCC 1015, harboring 19 INDELs.

**TABLE 2:** Genetic variants occurring in selected SM biosynthetic cluster regions in JSC-093350089 relative to ATCC 1015.

Gene name	ATCC 1015 gene (ASPNIDRAFT2_)	CBS 513.88 gene	JSC-093350089 variant	Mutation type	SM(s) produced
*pyrD*	1160356	An15g00470			Pyranonigrin A
*pyrC*	1154752	An18g00490	• Chr20.1_A99382AACC • Chr20.1_TCCA99386T • Chr20.1_TG99395T • Chr20.1_CA99512C • Chr20.1_C99516CT • Chr20.1_G99534GA • Chr20.1_TGC99735T • Chr20.1_A99738AAT • Chr20.1_T99840TA • Chr20.1_T99874TA • Chr20.1_AT99901A • Chr20.1_A99950AGC • Chr20.1_TAA99951T • Chr20.1_T99991TG • Chr20.1_AG100072A • Chr20.1_T100104TATC • Chr20.1_T100138TTAG • Chr20.1_AG100245A • Chr20.1_TATAG100617T • Chr20.1_G101007GA • Chr20.1_AAGG101585A	• Intergenic INDEL (downstream) • Intergenic INDEL (downstream) • Intergenic INDEL (downstream) • Intergenic INDEL (downstream) • Intergenic INDEL (downstream) • Intergenic INDEL (downstream) • Intergenic INDEL (downstream) • Intergenic INDEL (downstream) • Intergenic INDEL (downstream) • Intergenic INDEL (downstream) • Intergenic INDEL (downstream) • Intergenic INDEL (downstream) • Intergenic INDEL (downstream) • Intergenic INDEL (downstream) • Intergenic INDEL (downstream) • Intergenic INDEL (downstream) • Intergenic INDEL (downstream) • Intergenic INDEL (downstream) • Intergenic INDEL (downstream) • Intron INDEL • Intron INDEL	
*pyrB*	1188024	An18g00500			
*pyrE*	1139558	An18g00510			
pyrA*	1128344	An18g00520	Chr20.1_TGA121427T	Intergenic INDEL (downstream)	

*ktnA*	1222896	An04g09510	• Chr18.1_A6084T • Chr18.1_T6157C • Chr18.1_A6299G	• Intron SNP • Intron SNP • Intron SNP	Kotanin
*ktnB*	1126846	An04g09520			
ktnS*	1126849	An04g09530			
*ktnC*	1094747	An04g09540			
*ktnD*	1167408	An04g09540	• Chr18.1_C21654G • Chr18.1_G21655T • Chr18.1_T21665C • Chr18.1_GA23281G • Chr18.1_TGGACTTCATTGAC23400T • Chr18.1_AG23497A	• Synonymous SNP • Missense SNP • Missense SNP • Intergenic INDEL (upstream) • Promoter region INDEL • Intergenic INDEL (upstream)	

*epaC*	1114645	An09g01800	• Chr1.1_CT423438C • Chr1.1_T423443TA • Chr1.1_GC425206G	• Intergenic INDEL (downstream) • Intergenic INDEL (downstream) • Intergenic INDEL (upstream)	Pestalamides
*epaB*	1155683	An09g01820	Chr1.1_T426759TAA	3′ UTR INDEL	
orf3	1114655	An09g01840	Chr1.1_A430682AC	Intergenic INDEL (upstream)	
orf2	1080155	An09g01850	Chr1.1_AC432567A	Intergenic INDEL (downstream)	
epaA*	1080089	An09g01860	• Chr1.1_T436165G • Chr1.1_T439007G • Chr1.1_TC443237T • Chr1.1_GT443250G • Chr1.1_C443255CT	• Missense SNP • Missense SNP • Intergenic INDEL (downstream) • Intergenic INDEL (downstream) • Intergenic INDEL (downstream)	
orf1 (*azaJ*)	1114656	An09g01880	• Chr1.1_TTTA446289T • Chr1.1_C447318G • Chr1.1_CAA447488C • Chr1.1_A447492AAG • Chr1.1_T447561TG	• 3′ UTR INDEL • Synonymous SNP • 5′ UTR INDEL • 5′UTR INDEL • Intergenic INDEL (upstream)	

albA*	1099425	An09g05730	• Chr1.1_T1340832TTA • Chr1.1_C1346092T	• Intergenic INDEL (downstream) • Synonymous SNP	Aurasperones, fonsecinones, asperpyrones, and melanin

*Indicates core SM synthase gene (e.g., PKS, PKS-NRPS).

Within the kotanin biosynthetic gene cluster, several mutations were observed which may have influenced regulation and/or activity of *ktnD*, whose specific role in kotanin biosynthesis remains uncharacterized (Gil Girol et al., 2012). One synonymous and two missense SNPs were observed within *ktnD*, and 3 deletions were observed in the *ktnD* upstream intergenic region 339, 458, and 555 bps away from the 5′ untranslated region (UTR). Notably, the second of these deletions, which featured a loss of 13 bps, was predicted to be within the promoter region (genomic coordinates 23456–23311) using PromPredict, which predicts promoter regions based on DNA duplex stability and has been verified across a broad range of eukaryotic genomes (Rangannan and Bansal, 2010; Yella et al., 2018). Other variations occurring within the *ktn* cluster included 3 SNPs within the intron region of *ktnA*.

Many variants were observed within the pestalamide biosynthetic gene cluster, including two missense SNPs within *epaA*, whose gene product was proposed to perform the initial reaction in pestalamide biosynthesis (Wang et al., 2018). INDELs were observed within the 3′ UTR of *epaB*, and in both the 5′ and 3′ UTR of orf1. Additionally, intergenic INDELs were observed upstream of *epaC*, orf1, and orf3, and downstream of *epaA*, e*paC*, and orf2. One synonymous SNP was observed within PKS-encoding *albA*, along with a 2 bp insertion in the downstream intergenic region.

### Genetic Variant Analysis of SM Cluster Regulators

Next, we turned our attention to variations occurring in genes known to regulate the SMs that exhibited differential production in JSC-093350089, as variations in these genes may explain increased SM production levels. The transcription factor Fum21 has been reported to play a role in both pyranonigrin A and fumonisin production, with Fum21 deficiency resulting in a 25% reduction in pyranonigrin A production and complete elimination of fumonisin production (Aerts et al., 2018). Fum21 is regulated by FlbA, which is a regulator of G-protein signaling and plays a major role in sporulation (Krijgsheld et al., 2013). While the only variant associated with *fum21* was a downstream intergenic INDEL, several genetic variants within the *flbA* promoter (genomic coordinates 815849-815999) were observed, including a 16 bp insertion, a 7 bp deletion, and a 6 bp deletion (Table 3). Our investigation also revealed one splice region insertion and two intergenic INDELs downstream of *flbA*.

**TABLE 3:** Genetic variants occurring in selected SM regulators in JSC-093350089 relative to ATCC 1015.

Gene name	ATCC 1015 gene (ASPNIDRAFT2_)	CBS 513.88 gene	JSC-093350089 variant	Mutation type	SMs regulated
*fum21*	225717	An01g06900	Chr4.1_TAACTCTACATCGTAG ACTATAATCTC1760977T	Intergenic INDEL (downstream)	Pyranonigrin A and fumonisins
*gcnE*	1149812	An01g08160	• Chr4.1_A2074326AT • Chr4.1_TC2075538T • Chr4.1_A2075689C	• Intergenic INDEL (downstream) • Intron INDEL • Intergenic SNP (upstream)	Pestalamides and aurasperones
*flbA*	1158125	An02g03160	• Chr10.1_CTTTTTT815881C • Chr10.1_TTTTTTTC815912T • Chr10.1_A815964ATTTTTT TTTTACCCAT • Chr10.1_C816876CT • Chr10.1_C818639CT • Chr10.1_TA819635T	• Promoter region INDEL • Promoter region INDEL • Promoter region INDEL • Splice region INDEL • Intergenic INDEL (downstream) • Intergenic INDEL (downstream)	Pyranonigrin A and fumonisins via Fum21
*azaR*	1079852	An09g01870	• Chr1.1_ATAC443324A • Chr1.1_T443371TATC • Chr1.1_T444140G • Chr1.1_T445465TC	• Intergenic INDEL (downstream) • Intergenic INDEL (downstream) • Synonymous SNP • Intergenic INDEL (upstream)	Pestalamides

We then searched for variations within and/or nearby *gcnE*, which encodes a histone acetyltransferase responsible for the repression of several SMs in FGSC A1279, a SM-silent strain of *A. niger*, including the pestalamides and *albA* pathway naphtho-γ-pyrones (Wang et al., 2018). Our analysis revealed an upstream intergenic SNP 26 bps away from the CDS start codon, as well as a deletion in the intron region and an insertion in the downstream intergenic region (Table 3). Finally, we turned our attention to *azaR*, which is harbored within the pestalamide biosynthetic gene cluster and encodes a pathway-specific transcription factor (Zabala et al., 2012). We identified an insertion 222 bps away from the CDS start codon, along with a synonymous SNP and 2 downstream intergenic INDELs (Table 3).

### Assessment of the UV Resistance Potential of Pyranonigrin A

**FIGURE 3:**
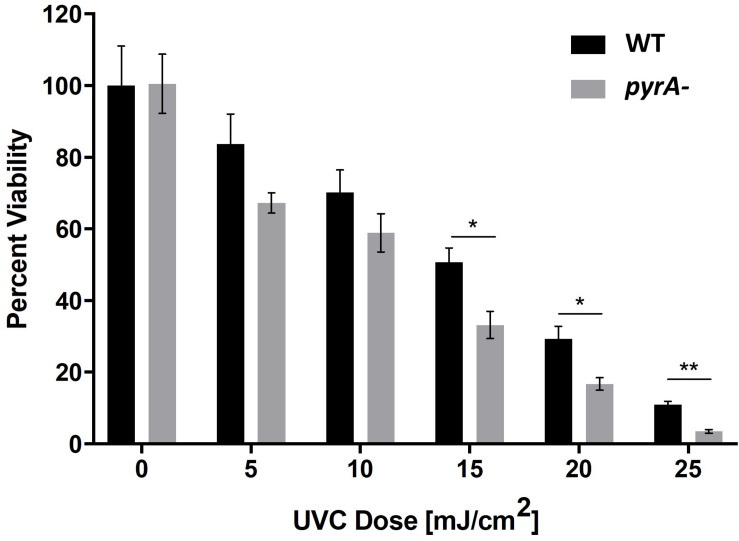
Percent survival following exposure to varying doses of UVC radiation for JSC-093350089 WT and JSC-093350089 *pyrA-* (CW12005). *P ≤ 0.05; **P ≤ 0.01.

We hypothesized that the enhanced production levels of pyranonigrin A in the ISS isolate played a role in protecting the strain from the high levels of radiation present in the spacecraft. This hypothesis was evaluated by comparing the UV sensitivity of pyranonigrin A-producing JSC-093350089 to pyranonigrin A-deficient JSC-093350089. It has been reported that *kusA* deletion significantly enhances the sensitivity of *A. niger* to UV exposure (Meyer et al., 2007). Therefore, to investigate whether pyranonigrin A confers UV resistance to *A. niger*, the *kusA* gene was first reintegrated into the *pyrA-* deletion strain (CW12005) to generate CW12015. Next, the JSC-093350089 WT and *pyrA-* strains were exposed to varying doses of UVC radiation ranging from 5–25 mJ/cm^2^ in triplicate. The results indicated that pyranonigrin A deficiency significantly reduces the viability of UV-exposed strains at doses greater than 15 mJ/cm^2^ (Figure 3). The effect became more pronounced as the radiation dose increased, with an approximate viability reduction of 34% observed at 15 mJ/cm^2^ (*P* = 0.005), 43% observed at 20 mJ/cm^2^ (*P* = 0.005), and 68% observed at 25 mJ/cm^2^ (*P* = 0.0002).

## Discussion

Although the persistence of *A. niger* in spacecraft (Pierson et al., 2013; Checinska et al., 2015) has been well-documented, few studies have investigated how spacecraft conditions alter its SM production. Metabolomic characterization combined with genetic analysis of *A. niger* strains that have inhabited spacecraft can provide valuable information about SM-based adaptation mechanisms of fungi capable of surviving in such environments. Additionally, despite a substantial effort to map fungal SMs to their biosynthesis genes, most clusters remain unlinked to their final product, which, in many cases, is due to the low production levels of many metabolites. There is therefore significant potential in analyzing SM production in fungal species isolated from various extreme environments, as such conditions may naturally optimize production yields of useful SMs, thereby reducing the cost of laborious laboratory-based optimization.

Metabolomic analysis of the *A. niger* laboratory strain and ISS isolate revealed significant differences in SM production levels. This finding is not surprising given the unique stresses associated with spaceflight and the fact that SM production is an evolutionary-derived trait to confer selective advantage in distinct ecological niches (Brakhage, 2013). While a recent study found that growth in ISS conditions is capable of altering SM production in *Aspergillus nidulans* (Romsdahl et al., 2019), the metabolomic changes encountered in JSC-093350089, which are conserved on Earth, are likely the result of a substantial changes at the genomic or epigenomic level, which may have been selected for by pressures associated with ISS conditions, such as microgravity, enhanced radiation, and low nutrient availability. Interestingly, the spaceflight environment appeared to select for a strain that produces enhanced levels of therapeutically relevant SMs, including the antioxidant pyranonigrin A (Miyake et al., 2007), the human cancer cytotoxic agent bicoumanigrin A (Hiort et al., 2004), the antimicrobial aurasperone A (Shaaban et al., 2012), and the antifungal and antioxidant aurasperone B (Choque et al., 2015).

Perhaps the most significant observation was enhanced production of pyranonigrin A in JSC-093350089 relative to ATCC 1015, which only produces the SM at basal levels. On the ISS, radiation capable of penetrating the spacecraft generates reactive oxygen species (ROS) within biological systems (Gabani and Singh, 2013). Oxidative stress occurs when ROS overwhelm an organism’s antioxidant defense mechanisms, resulting in the generation of oxidative damage among DNA, proteins, lipids, and other vital cell components (Lehnert and Iyer, 2002; Gabani and Singh, 2013). Antioxidants have enormous therapeutic potential, as they neutralize the harmful effects of ROS, which can cause or exacerbate a range of human diseases, including cancer, diabetes, cardiovascular, and neurodegenerative diseases (Firuzi et al., 2011). Examination of metabolic reserves of fungi isolated from enhanced radiation environments therefore provides a means of identifying fungal strains that produce optimized levels of specific therapeutics, as illustrated by this study. Further, investigation into the underlying genetics that may be responsible for increased production of bioactive compounds provides a useful starting point for future production yield optimization efforts.

We suspected that the conserved increase in some SM production levels may be due to variations within genomic regions that commonly possess regulatory elements, such as the promoter, the 5′ UTR, or the 3′ UTR. This was investigated by performing a comparative genetic analysis of SM clusters and regulators. This initially required identification of the pyranonigrin A biosynthetic gene cluster in *A. niger*, which has a different genetic architecture than the cluster identified in *P. thymicola* (Supplementary Figure S9; Tang et al., 2018). While confirming the involvement of these genes in *A. niger*, we identified an additional cluster gene, *pyrE*, that was previously reported to not be involved in pyranonigrin A biosynthesis.

Unexpectedly, we did not identify any variants within the *pyr* cluster that could clearly explain the significant increase in pyranonigrin A production in the ISS strain. We therefore extended our study to include genes encoding products known to regulate pyranonigrin A. Thus far, this is limited to the transcription factor Fum21, which is regulated by FlbA, a regulator of G-protein signaling that plays a critical role in sporulation and whose deficiency results in thin cell walls and cell lysis (Krijgsheld et al., 2013; Aerts et al., 2018). It has been reported that Fum21 deficiency leads to downregulation of genes from 4 different SM clusters, accompanied by a 25% reduction in pyranonigrin A production and complete elimination of fumonisin production (Aerts et al., 2018). Although we did not identify any critical mutation within/near *fum21*, our investigation revealed 3 INDELs that account for the modification of 29 bps within the promoter region of *flbA*. It is highly probable that these mutations could lead to altered regulation of FlbA, which may help explain the dramatic increase in pyranonigrin A production in the ISS strain. Further experiments should be conducted to confirm this possibility, and to explore the potential impact of the observed promoter-region mutations on other biological processes within *A. niger*, such as sporulation and cell wall structure, as such studies may provide insight into why these variations may be favorable in ISS conditions. On the other hand, increased pyranonigrin A production levels may also be the result of differences at the epigenetic level, which would be an interesting avenue to explore but was outside the scope of this investigation.

Second only to pyranonigrin A, kotanin exhibited a 10-fold increase in production levels in JSC-093350089. Within its biosynthetic gene cluster, mutations were observed within *ktnD* that could have altered both its regulation and activity, including a 13 bp deletion within the promoter region and 2 missense mutations. It is unclear whether altered regulation of *ktnD* could explain the increase in kotanin production levels, as the specific role of KtnD, a monooxygenase, in the biosynthesis of kotanin has not been determined, and KtnS was proposed to be the rate-limiting step in the pathway (Gil Girol et al., 2012).

Within the pestalamide biosynthetic cluster, several mutations were observed which may explain its twofold increase in production in the ISS strain. Two missense mutations were observed within the gene encoding EpaA, the NR-PKS proposed to perform the initial reaction in pestalamide biosynthesis. Notably, INDELs were observed within the 5′ UTR and 3′ UTR of orf1. UTRs often contain regulatory elements that play key roles in appropriate expression of a gene, and therefore the observed variation may be responsible for altered regulatory patterns of orf1, which encodes for an oxidoreductase. The gene product of orf1, also known as AzaJ, is also involved in the biosynthesis of the azanigerones, which are produced by the same cluster as the pestalamides (Zabala et al., 2012). In azanigerone biosynthesis, AzaJ is proposed to catalyze the oxidation of an aldehyde to a carboxylic acid, partially facilitating the transformation of azanigerone C to azanigerone A. In pestalamide biosynthesis, AzaJ was proposed to be involved in the generation of the intermediate carbonarone A from the polyketide precursor, which involves oxidation and transamination (Wang et al., 2018). Further studies need to be performed to determine if this step in the pathway is rate-limiting, and if altered expression of *azaJ* could be responsible for the increase in pestalamide production levels. Another potentially significant mutation that we observed was an insertion in the 3′ UTR of *epaB*, which encodes an acyl:CoA ligase that may catalyze the formation of 2-methylsuccinyl-CoA, as it plays a similar role in the azanigerone pathway. 2-methylsuccinyl-CoA is then used as a substrate by EpaC followed by cyclization to form pestalamide. Further experiments should be performed to confirm whether the observed 3′ UTR variant alters expression levels of *epaB*, which then leads to altered pestalamide production levels.

Next, we investigated genetic variants associated with genes encoding known regulators of pestalamide, including the pathway-specific transcription factor AzaR and the epigenetic regulator GcnE (Zabala et al., 2012; Wang et al., 2018). However, this analysis did not reveal any substantial findings; while both genes possess upstream intergenic variants in JSC-093350089, they were predicted to outside the promoter region.

It is reasonable to presume that enhanced production of molecules that confer oxidative stress resistance is a key adaptive characteristic of fungi inhabiting high-radiation environments, as this has been observed among other species (Baqai et al., 2009; Robinson et al., 2011). This may explain the enhanced production levels of pyranonigrin A in the ISS isolate, and is supported by the UV resistance study, which suggests that pyranonigrin A plays a role in conferring radiation resistance in *A. niger*. Additionally, we observed a cumulative increase in the production of naphtho-γ-pyrones, which are produced by AlbA, the same PKS responsible for 1,8-dihydroxynaphthalene-melanin (DHN-melanin) biosynthesis. This stays in agreement with the observed enrichment of AlbA in the proteome of JSC-093350089 relative to ATCC 1015 (Romsdahl et al., 2018). Such observations suggest that the ISS isolate produces enhanced levels of DHN-melanin, and is consistent with previous reports that fungi inhabiting high-radiation environments produce enhanced levels of melanin (Singaravelan et al., 2008). In microbial systems, melanin functions as a UV-protectant, an antioxidant, a thermoregulator, and as a toxin-sequestering agent (Cordero and Casadevall, 2017). It is therefore reasonable to presume that increased melanin production may be an adaptive feature that is selected for by spaceflight environments. We were unable to identify any mutations associated with *albA* that may explain its upregulation and the associated increase in naphtho-γ-pyrone production. Additionally, we could not investigate variants occurring in naphtho-γ-pyrone tailoring enzymes, as those genes are not clustered with *albA* and have not yet been characterized. Further, as discussed before with regard to the pestalamides, while production levels of naphtho-γ-pyrones were reported to increase following deletion of the epigenetic regulator *gcnE*, we did not identify any mutations associated with *gcnE* that may explain increased production levels of naphtho-γ-pyrones.

In summary, this investigation revealed the distinct metabolomic fingerprint of a melanized fungal species capable of withstanding ISS conditions, which featured enhanced production of a molecule with antioxidant and UV-protective properties. Genetic analysis revealed variants that may be responsible for the observed increase in SM production levels. These findings illustrate the therapeutic and economic potential associated with investigating metabolite production in microbes isolated from extreme environments and provide a framework for future efforts to genetically optimize production yields of the bioactive SMs described in this study.

## Data Availability Statement

All datasets generated for this study are included in the article/Supplementary Material.

## Author Contributions

JR drafted the manuscript, contributed to sample processing, and was responsible for data analysis and interpretation. AB contributed to sample processing and data interpretation. Y-MC contributed to secondary metabolic analysis and interpretation. KV and CW designed the study, interpreted the data, and drafted the manuscript. All authors read and approved the final manuscript.

## Conflict of Interest

The authors declare that the research was conducted in the absence of any commercial or financial relationships that could be construed as a potential conflict of interest.

## Footnotes

1 https://broadinstitute.github.io/picard/
