# Supplementary material for: Metabolomic Analysis of Aspergillus niger Isolated From the International Space Station Reveals Enhanced Production Levels of the Antioxidant Pyranonigrin A
Source: Front Microbiol. 2020 May 21;11:931. doi: 10.3389/fmicb.2020.00931 (PMC7326050; doi:10.3389/fmicb.2020.00931)
Supplement: Supplementary file 1 [file Data_Sheet_1.pdf]

Supplementary Material

Frontiers in Microbiology

Metabolomic analysis of *Aspergillus niger* isolated from the International Space Station reveals enhanced production levels of the antioxidant pyranonigrin A

Jillian Romsdahl[1], Adriana Blachowicz[1,2], Yi-Ming Chiang[1], Kasthuri Venkateswaran[2], and Clay C.C. Wang[1,3*]
[1]Department of Pharmacology and Pharmaceutical Sciences, School of Pharmacy, University of Southern California, Los Angeles, California 90089, United States
[2] Biotechnology and Planetary Protection Group, Jet Propulsion Laboratory, California Institute of Technology, Pasadena, CA
[3] Department of Chemistry, Dornsife College of Letters, Arts, and Sciences, University of Southern California, Los Angeles, California 90089, United States

*** Correspondence:**

Clay C.C. Wang
School of Pharmacy
University of Southern California
1985 Zonal Ave Rm 406A
Los Angeles, CA, 90089-9121
Phone: (323) 442-1670
Email: clayw@usc.edu

Table of Contents

Table S1. *Aspergillus niger* strains used in this study.

Table S2. Primers used in this study.

Table S3. Comparison of *A. niger* PKS-NRPSs to pyranonigrin E-producing PKS-NRPS.

Figure S1. Results of diagnostic PCR for JSC-093350089 mutant strains.

Figure S2. Strategy for gene deletion via selection marker replacement.

Figure S3. Strategy for *pyrG* deletion.

Figure S4. Strategy for *kusA* reintegration.

Figure S5. UV-Vis and ESIMS (positive or negative mode) spectra of compounds identified in this study.

Figure S6. Verification of pyranonigrin A production in JSC-093350089.

Figure S7. Individual secondary metabolite production of AlbA pathway SMs in JSC-093350089 compared to ATCC 1015.

Figure S8. DAD total scan and MS extracted ion chromatogram at *m/z* 224 of extracts from JSC-093350089 WT and mutant strains *pynA*- and *pyrA*-.

Figure S9. Comparison of gene distribution for pyranonigrin A biosynthesis cluster in *P. thymicola* and *A. niger*.

Table S1. *Aspergillus niger* strains used in this study

Strain #	Parent strain	Introduced mutation	Genotype
ATCC 1015	ATCC 1015	WT	None (WT)
JSC-093350089	JSC-093350089	WT	None (WT)
CW12002	JSC-093350089	*kusA*- (An15g02700)	*kusA*::*hph*
CW12003	CW12002	*pyrG*- (An12g03570)	*kusA*::*hph*; *pyrG*-
CW12004	CW12003	*pynA*- (An11g00250)	*kusA*::*hph*; *pyrG*-; *pynA*::*AfpyrG*
CW12005	CW12003	*pyrA*- (An18g00520)	*kusA*::*hph*; *pyrG*-; *pyrA*::*AfpyrG*
CW12006	CW12003	*albA*- (An09g05730)	*kusA*::*hph*; *pyrG*-; *albA*::*AfpyrG*
CW12007	CW12006	*AfpyrG*-	*kusA*::*hph*; *pyrG*-; *albA*-
CW12008	CW12007	An18g00480-	*kusA::hph; pyrG-; albA-;* An18g00480*::AfpyrG*
CW12009	CW12007	*pyrC*- (An18g00490)	*kusA::hph; pyrG-; albA-; pyrC::AfpyrG*
CW12010	CW12007	*pyrB*- (An18g00500)	*kusA::hph; pyrG-; albA-; pyrB::AfpyrG*
CW12011	CW12007	*pyrE*- (An18g00510)	*kusA::hph; pyrG-; albA-; pyrE::AfpyrG*
CW12012	CW12007	*pyrA*- (An18g00520)	*kusA::hph; pyrG-; albA-; pyrA::AfpyrG*
CW12013	CW12007	An18g00530-	*kusA::hph; pyrG-; albA-;* An18g00530*::AfpyrG*
CW12014	CW12005	*AfpyrG*-	*kusA*::*hph*; *pyrG*-; *pyrA*-
CW12015	CW12014	+ *kusA*	*pyrG*-; *pyrA*-; *AfpyrG-kusA*

Table S2. Primers used in this study (5' → 3')

kusA (An15g02700) deletion construct	
kusA_F1	GGCCGAGAACAAGAGAACCA
kusA_F2	CGTTTCCGTTTCCTCGCTTG
kusA_R3	CGGTGAGTTCAGGCTTTTTCATTAACCAGGAACAAGTGGGGC
kusA_F4	GTCCGAGGGCAAAGGAATAGGCCTGAGGACATGAGCTTGT
kusA_R5	GTAGTGGCCGTGTCATGGAA
kusA_R6	ACGACCACGAGAGGACTACA
kusA_DFw	CATCACCGCATGCACTGTTG
kusA_DRev	GCACGTGACGGAAGAAGTCT
hph gene	
hph Fw	GCTGGAGCTAGTGGAGGTC
hph Rev	CGGTCGGCATCTACTCTATT
pyrG (An12g03570) deletion construct	
PyrG_F1	TGTGCCAGTCAATTGTCCGA
PyrG_F2	CTCCTCATCCACCGTCATCG
PyrG_R3	CTTTGCAGGTGTGGCTGAACCGGTATTGATCCTGCAGGCT
PyrG_F4	GTTCAGCCACACCTGCAAAG
PyrG_R5	CTGTACCATCAGCGCTCCTC
PyrG_R6	GCAAGCGAAGTATGGCAGTG
Af_PyrG_Fw	CAATGCTCTTCACCCTCTTCG
Af_PyrG_Rev	CTGAGAGGAGGCACTGATGC
pynA (An11g00250) deletion constructs	
pynA_F1	ATCGCAGCAATTTCCATGCC
pynA_F2	ACAAGGTGATGGTCCGGTTC
pynA_R3	CGAAGAGGGTGAAGAGCATTGTTTGGGCCAAATTGCGAACC
pynA_F4	GCATCAGTGCCTCCTCTCAGCTGAGGATGGGGGCAGAATC
pynA_R5	CATTGCCTTCTCGACCCTGT
pynA_R6	GGCTGCACTAAGCTGTGGTA
pyrA (An18g00520) deletion constructs	
pyrA_F1	CATGTCCTATTCGACCCGGG
pyrA_F2	TCGGTTCAGACCCCGAGTA
pyrA_R3	CGAAGAGGGTGAAGAGCATTGGGGTGGTGAGGGAGGAAAAG
pyrA_F4	GCATCAGTGCCTCCTCTCAGAGCCTTGTTGTCGTCCACAA
pyrA_R5	TTCCACGACAGCCACATTGT
pyrA_R6	CATCGGCCCATTTCTGCATG
pyrA_R3_ PyrG_recycle	TTGTGGACGACAACAAGGCTGGGTGGTGAGGGAGGAAAAG
pyrA_F4_PyrG_recycle	AGCCTTGTTGTCGTCCACAA
albA (An09g05730) deletion constructs	
albA_F1	AGTGCAGAGTCGAGTCGAAC
albA_F2	CAAATGAACCGGCCATGCTC
albA_R3	TGACCTCCACTAGCTCCAGCCCTTCCACATCCGTGTCGAT

albA_F4	AATAGAGTAGATGCCGACCGATCAGTGCCCATGCCCAATT
albA_R5	CCCTGAAACGGAAGGTCGAA
albA_R6	CATCGCTAGAACGCAAAGCC
alba_R3_PyrG_recycle	AATTGGGCATGGGCACTGATCCTTCCACATCCGTGTCGAT
alba_F4_PyrG_recycle	ATCAGTGCCCATGCCCAATT
An18g00480 deletion constructs	
An18g00480_F1	TCGAACTGGACAGTGCTGAC
An18g00480_F2	GATGGGAGGACACTATGCCG
An18g00480_R3	CGAAGAGGGTGAAGAGCATTGCCGCTTCCTCCCAATTTCCT
An18g00480_F4	GCATCAGTGCCTCCTCTCAGATTGTGAGGCAGCCATTCGA
An18g00480_R5	CTTGCCTTCTCCTATGCCCC
An18g00480_R6	TTGAAGACGTGGGGGAGTTG
pyrC (An18g00490) deletion constructs	
pyrC_F1	GGTGCTACCGCTGGTATACC
pyrC_F2	CGTATCCGAAGTACAGCGCT
pyrC_R3	CGAAGAGGGTGAAGAGCATTGGACCGGACTGATGGTGTGAG
pyrC_F4	GCATCAGTGCCTCCTCTCAGGCTTGATTGGGCTTTGGGTG
pyrC_R5	GGTCCCCGAAAACTGGGTAG
pyrC_R6	TCGGCAGTCATTCCAAACGA
pyrB (An18g00500) deletion constructs	
pyrB_F1	CGGCCAAGAGGTGAGGATAC
pyrB_F2	GTTGGCGAATTGGGCTCATC
pyrB_R3	CGAAGAGGGTGAAGAGCATTGCAATGGCCCTTACCACCCTT
pyrB_F4	GCATCAGTGCCTCCTCTCAGAACGAGGGTTGAAGCGAGAG
pyrB_R5	TGACAGAGTGCGGAAAGACC
pyrB_R6	TGACAAGGCCCTTCTTCGAC
pyrE (An18g00510) deletion constructs	
pyrE_F1	GAAGCCAACTACCAGCGAGT
pyrE_F2	GCTCACCTGACACTTCGACA
pyrE_R3	CGAAGAGGGTGAAGAGCATTGGACAAGGCCCTTCTTCGACA
pyrE_F4	GCATCAGTGCCTCCTCTCAGCCTCCCTCAAGTTACGCGTT
pyrE_R5	GTTGGTCTGGCGCATTCATC
pyrE_R6	CAGTCGTTGTTGGGGATCCA
An18g00530 deletion constructs	
An18g00530_F1	CCCGTCAATTATCTCGCGGA
An18g00530_F2	TGCACGACACTACTCAACCC
An18g00530_R3	CGAAGAGGGTGAAGAGCATTGACGGTCGTTGTCTTGTCTCC
An18g00530_F4	GCATCAGTGCCTCCTCTCAGTTGCTTGGAGCGAAGACGAT
An18g00530_R5	CAAGTTGCACGGAGGTAGGT
An18g00530_R6	GCAAGACGCTGTTGACACTG
kusA (An15g02700) reintegration construct	
kusA_reint_F1	AAAAGACCGTTCGTATCGCG

kusA_reint_R3	CGAAGAGGGTGAAGAGCATTGCTTCCTTTCGGCGCTCTCTT
kusA_reint_F4	GTCCGAGGGCAAAGGAATAGGCCTGAGGACATGAGCTTGT
kusA_reint_R6	ACGACCACGAGAGGACTACA

Table S3. Comparison of *A. niger* PKS-NRPSs to pyranonigrin E-producing PKS-NRPS

ATCC 1015 gene (JGI Designation)	CBS 513.88 gene (NCBI Designation)	% Identity	% Subject Coverage
Aspni7:1128344	An18g00520	53.4	89.8
Aspni7:1188722	An08g03790	41.1	38.5
Aspni7:1112058	An11g06460	40.1	40.1
Aspni7:1170655	no homolog	41.2	36.6
Aspni7:1087173	no homolog	40.5	38.3
Aspni7:1122199	An02g08290	40.6	35.4
Aspni7:1099903	An14g01910	38.4	38.4
Aspni7:1115863	An14g04850	39.5	42.6

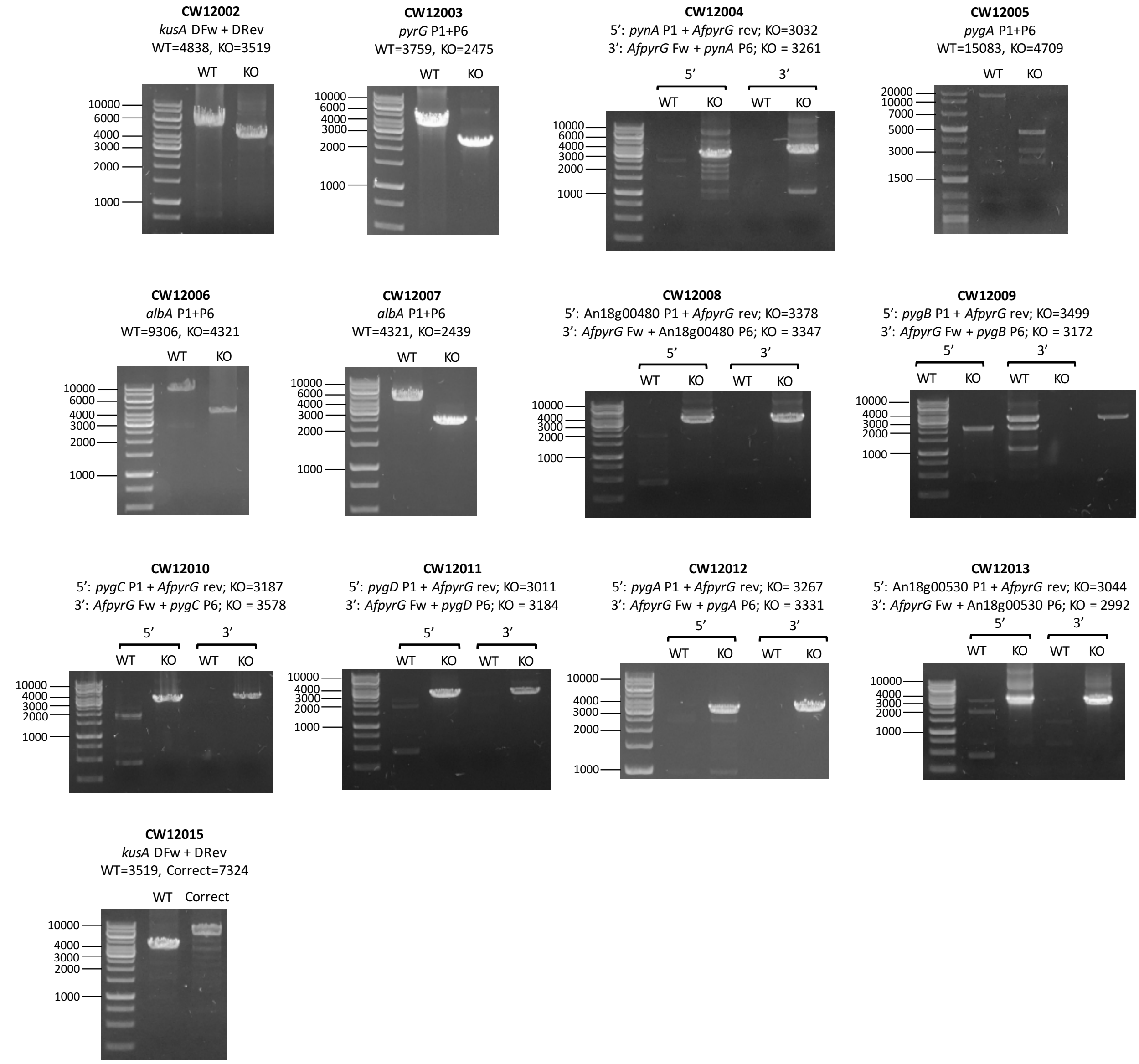

Figure S1. Results of diagnostic PCR for JSC-093350089 mutant strains.

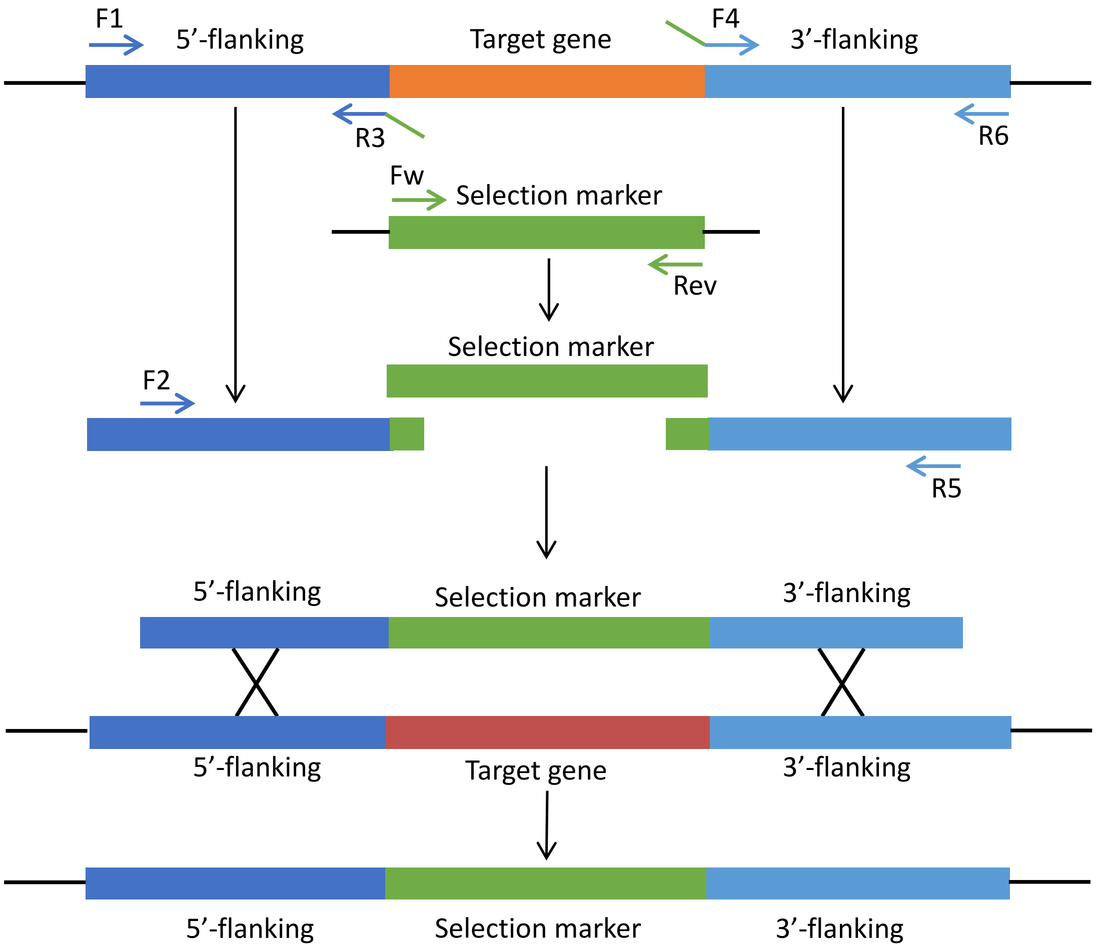

Figure S2. Strategy for gene deletion via selection marker replacement.

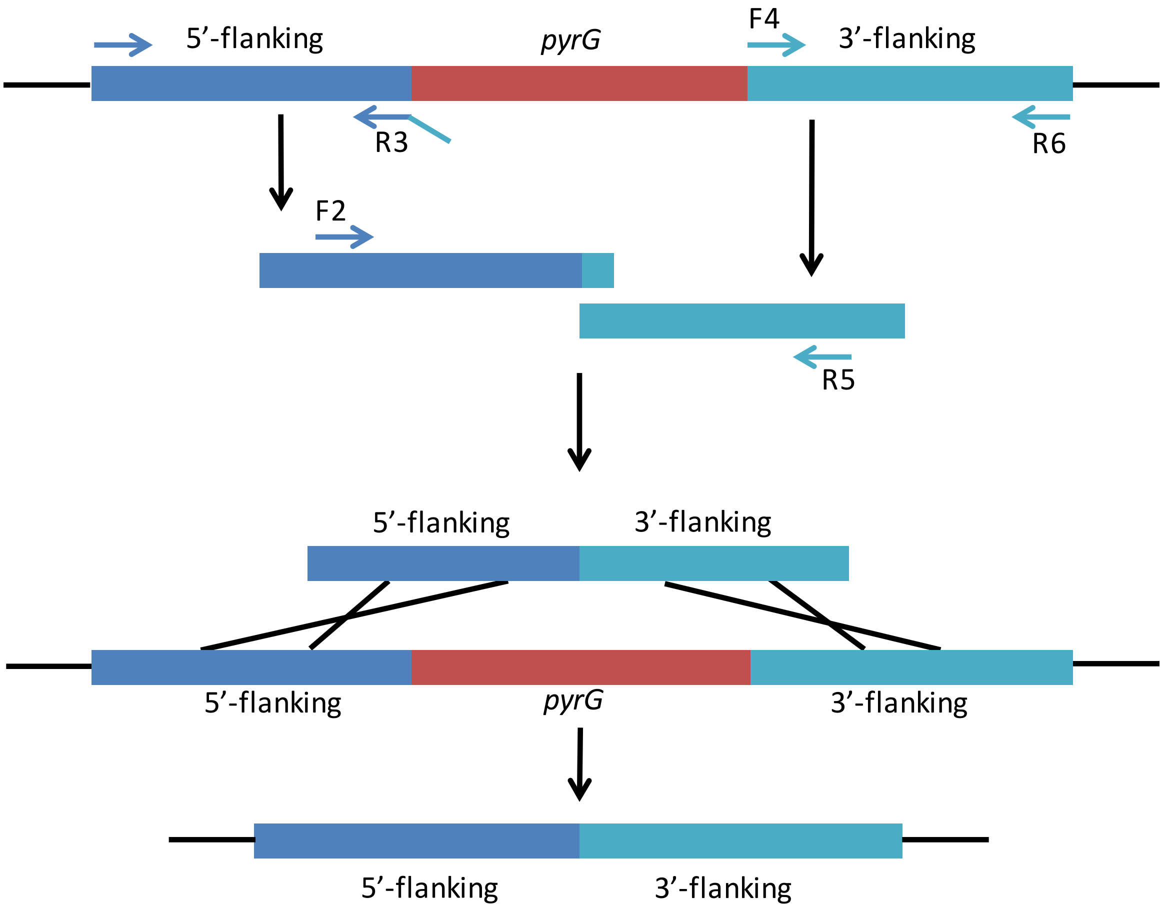

Figure S3. Strategy for *pyrG* deletion.

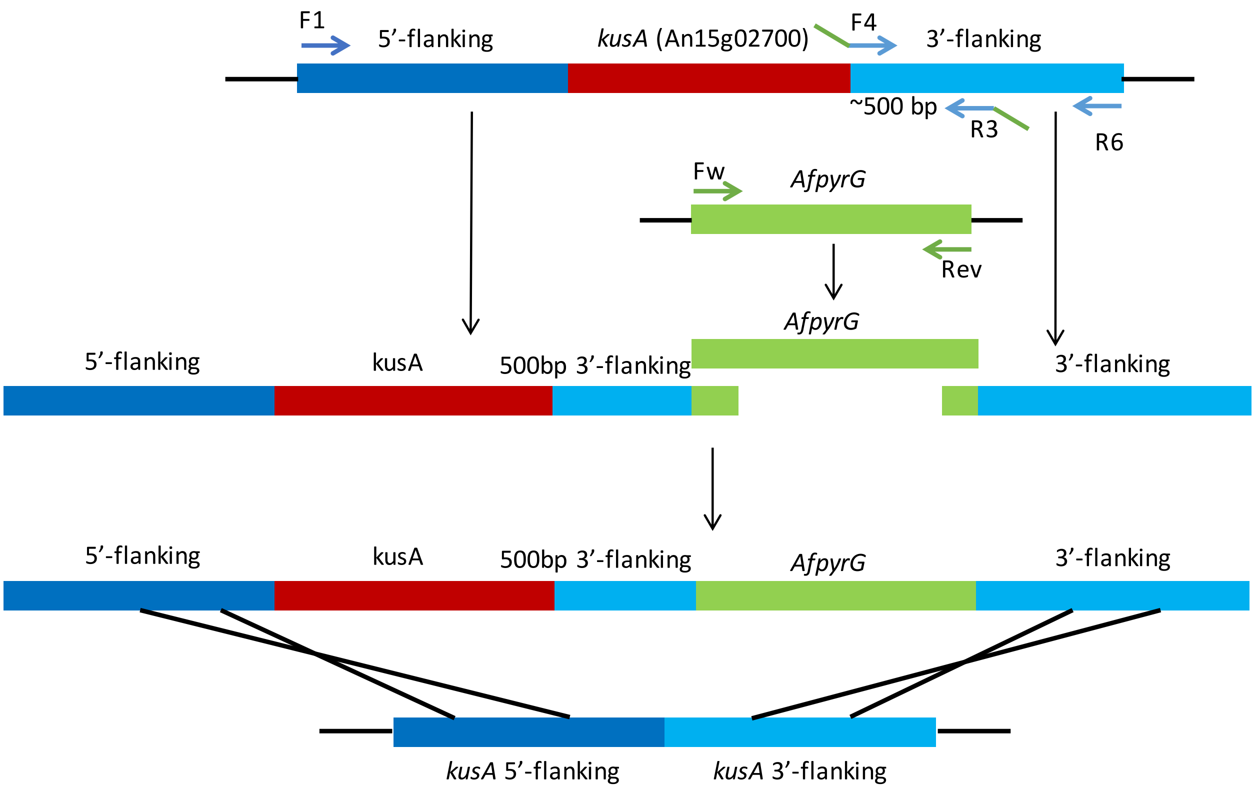

Figure S4. Strategy for *kusA* reintegration.

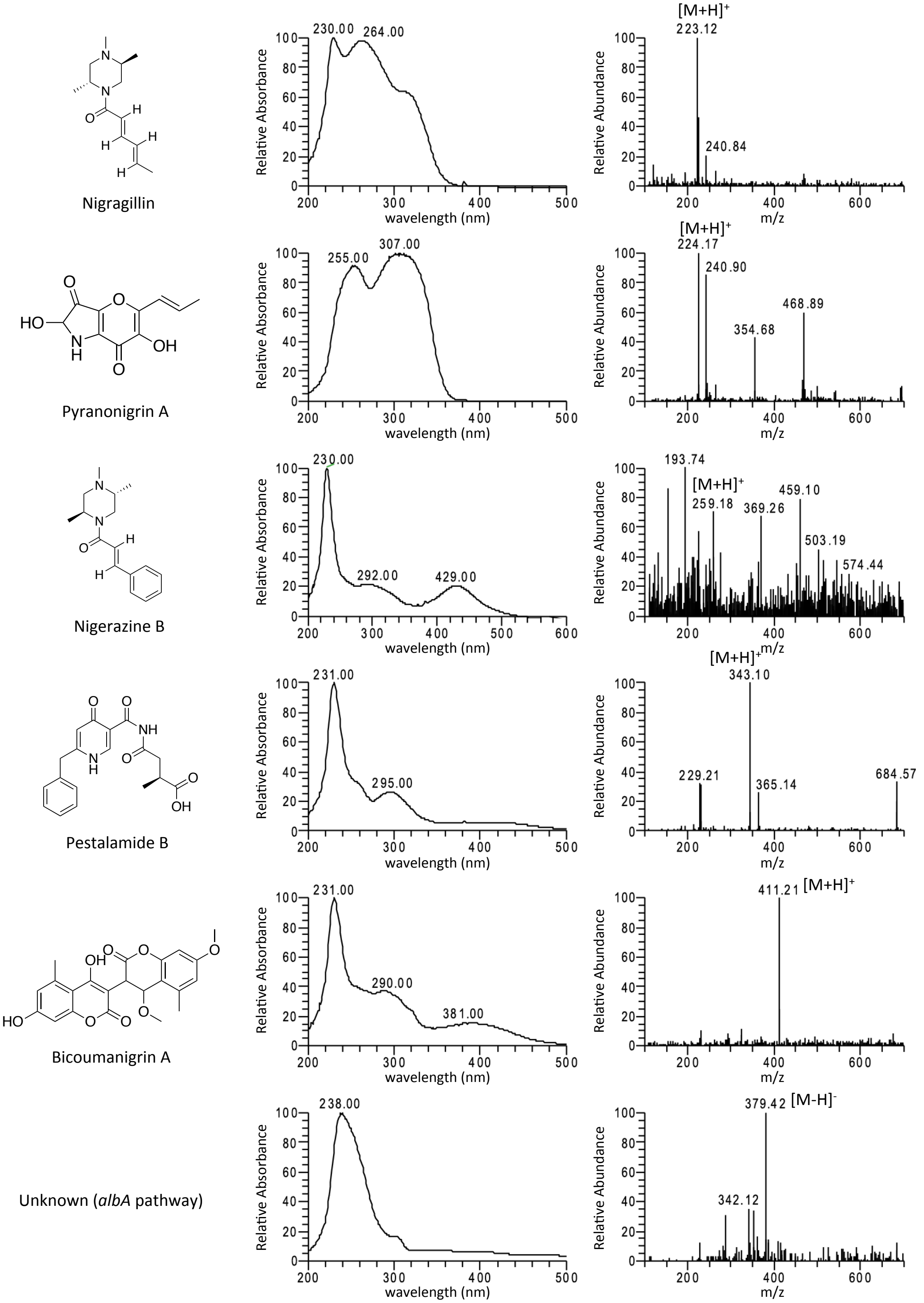
Nigragillin
Pyranonigrin A
Nigerazine B
Pestalamide B
Bicoumanigrin A
Unknown (*albA* pathway)
Relative Absorbance
wavelength (nm)
Relative Abundance
m/z
230.00
264.00
[M+H]+
223.12
240.84
255.00
307.00
224.17
240.90
354.68
468.89
292.00
429.00
193.74
259.18
369.26
459.10
503.19
574.44
231.00
295.00
343.10
229.21
365.14
684.57
290.00
381.00
411.21
238.00
379.42
[M-H]-
342.12

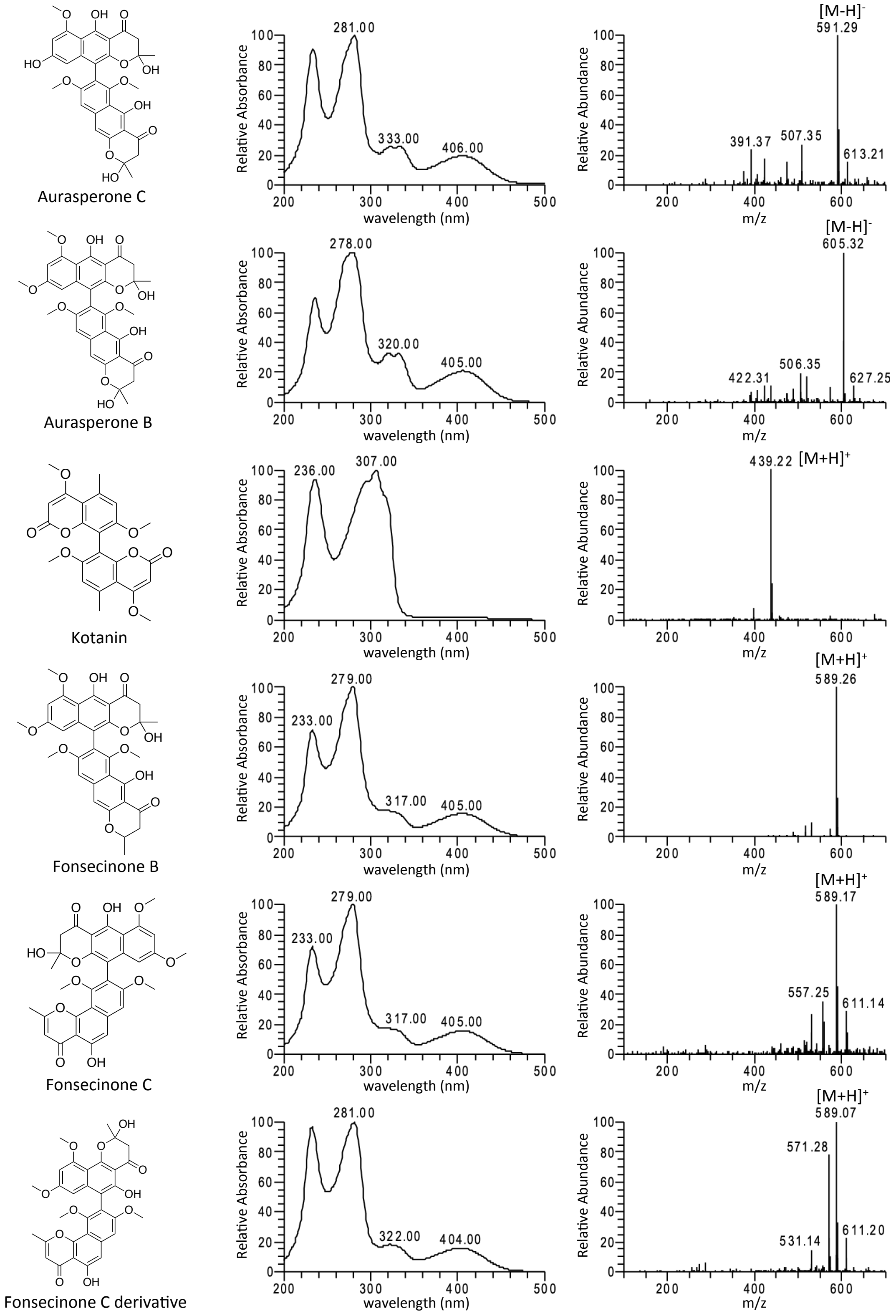

Aurasperone C
Aurasperone B
Kotanin
Fonsecinone B
Fonsecinone C
Fonsecinone C derivative
Relative Absorbance
wavelength (nm)
Relative Abundance
m/z
281.00
333.00
406.00
[M-H]-
591.29
391.37
507.35
613.21
278.00
320.00
405.00
605.32
422.31
506.35
627.25
236.00
307.00
[M+H]+
439.22
279.00
233.00
317.00
405.00
589.26
589.17
557.25
611.14
281.00
322.00
404.00
589.07
571.28
531.14
611.20

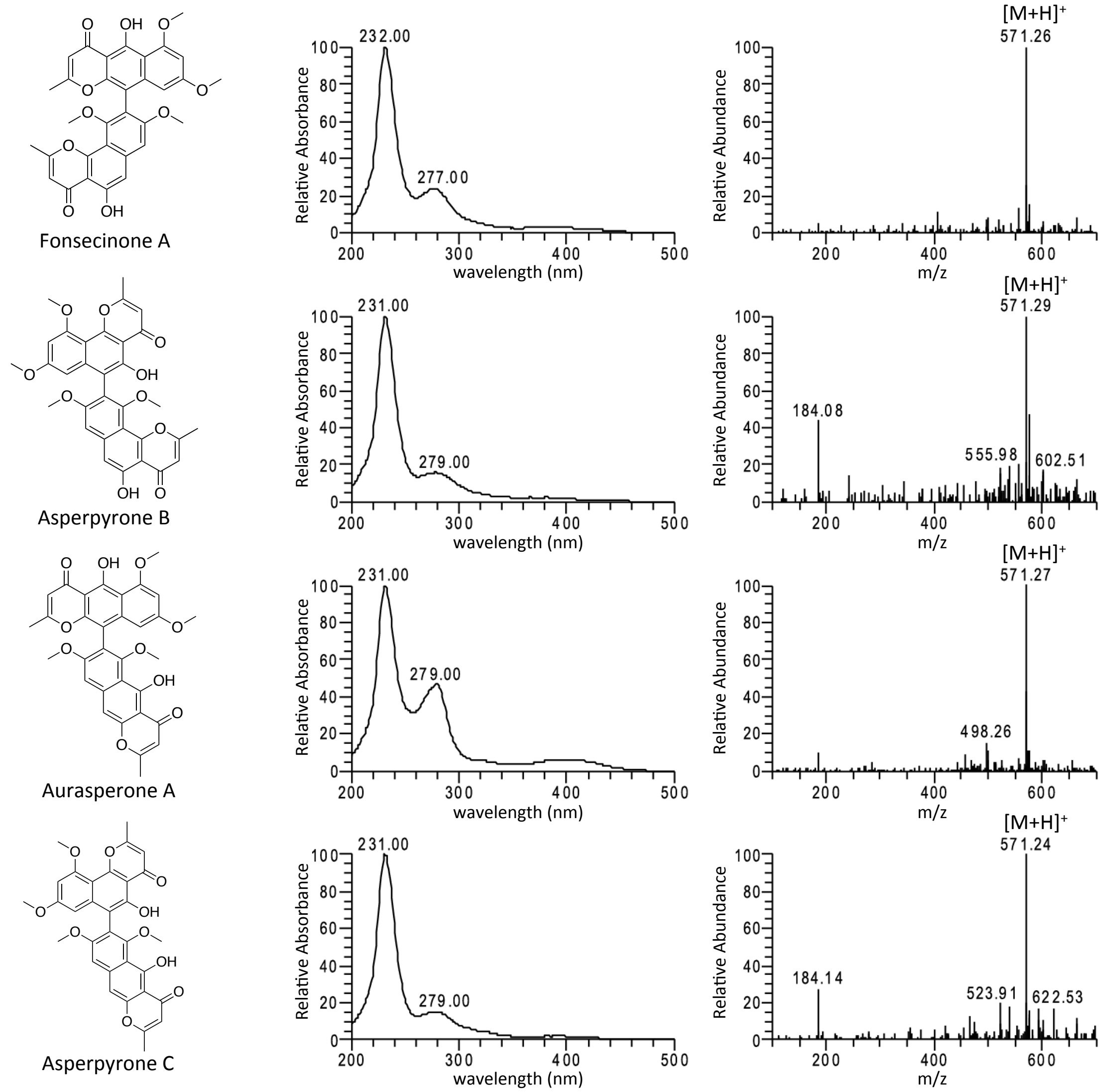

Figure S5. UV-Vis and ESIMS (positive or negative mode) spectra of compounds identified in this study.

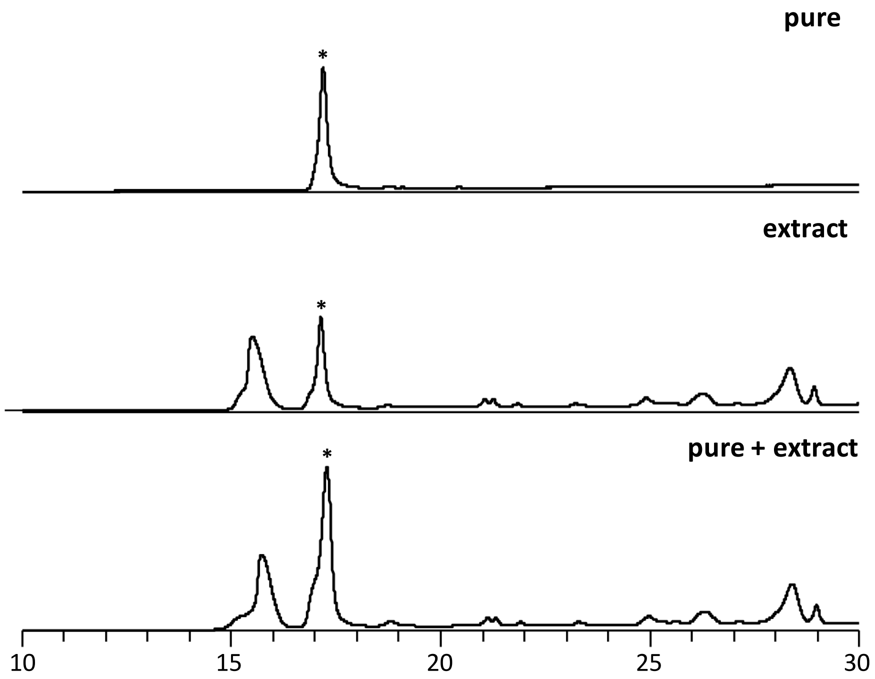

Figure S6. Verification of pyranonigrin A production in JSC-093350089. LC-MS profiles of pure pyranonigrin A (purchased from Enzo Life Sciences) and extract from JSC-093350089 following growth on glucose minimal media, as detected by UV total scan.

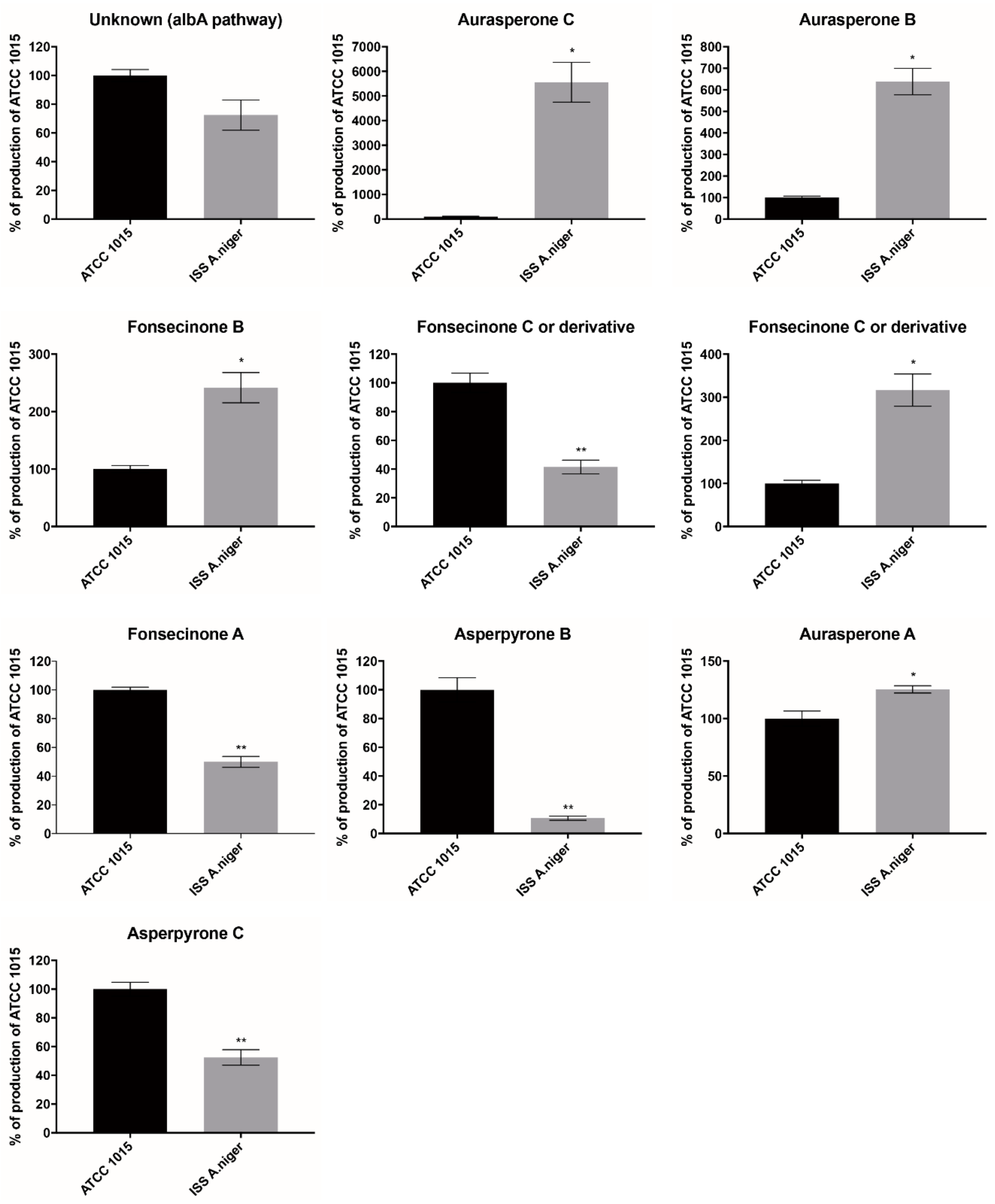

Figure S7. Quantification of *albA* pathway secondary metabolites showing percent change for metabolite production in JSC-093350089 compared to ATCC 1015. Significance was determined using Welch's t-test.

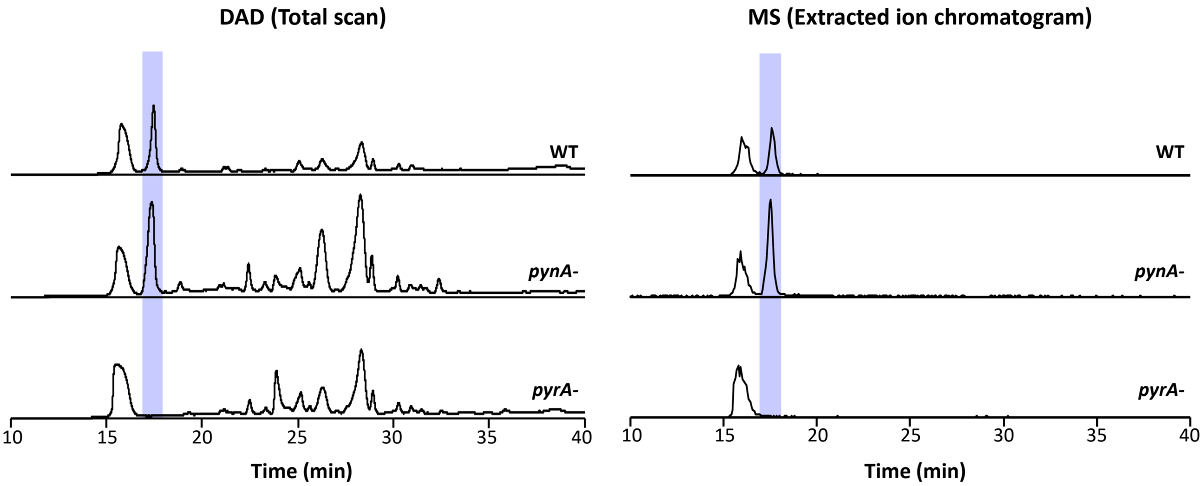

Figure S8. DAD total scan and MS extracted ion chromatogram at *m*/*z* 224 of extracts from JSC-093350089 WT and mutant strains *pynA*- and *pyrA*-. Highlighted peaks indicate pyranonigrin A production.

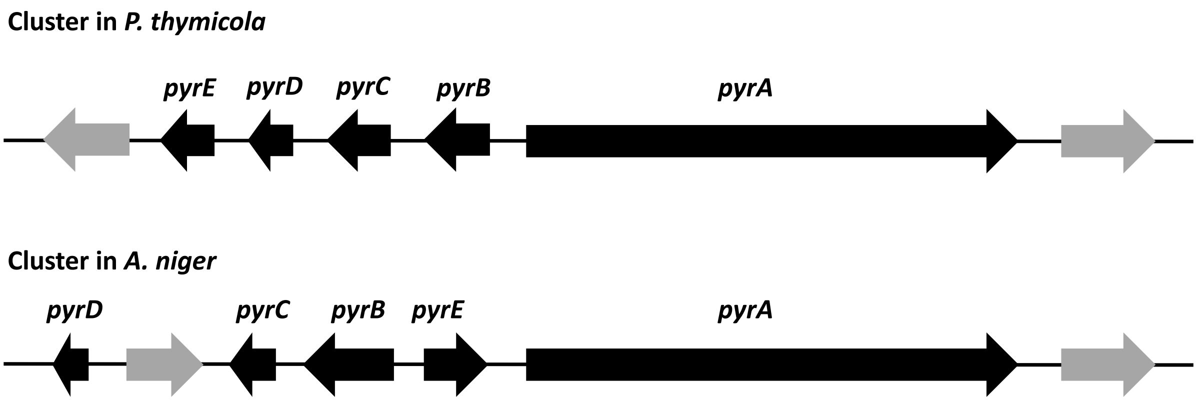

Figure S9. Comparison of gene distribution for pyranonigrin A biosynthesis cluster in *P. thymicola* and *A. niger*.
